# Optimization of fatty alcohol biosynthesis pathway for selectively enhanced production of C12/14 and C16/18 fatty alcohols in engineered *Escherichia coli*

**DOI:** 10.1186/1475-2859-11-65

**Published:** 2012-05-20

**Authors:** Yan-Ning Zheng, Ling-Ling Li, Qiang Liu, Jian-Ming Yang, Xiang-Wei Wang, Wei Liu, Xin Xu, Hui Liu, Guang Zhao, Mo Xian

**Affiliations:** 1 Qingdao Institute of Bioenergy and Bioprocess Technology, Chinese Academy of Sciences, No.189 Songling Road, Laoshan District, Qingdao, 266101, China; 2 Graduate University of Chinese Academy of Sciences, Beijing, 100049, China; 3 College of Food Science, Sichuan Agricultural University, Yaan, 625014, China

**Keywords:** Fatty alcohol, *Escherichia coli*, Pathway optimization, Selective production, Fermentation

## Abstract

**Background:**

With the increasing stress from oil price and environmental pollution, aroused attention has been paid to the microbial production of chemicals from renewable sources. The C12/14 and C16/18 alcohols are important feedstocks for the production of surfactants and detergents, which are widely used in the most respected consumer detergents, cleaning products and personal care products worldwide. Though bioproduction of fatty alcohols has been carried out in engineered *E. coli*, several key problems have not been solved in earlier studies, such as the quite low production of C16/18 alcohol, the lack of optimization of the fatty alcohol biosynthesis pathway, and the uncharacterized performance of the engineered strains in scaled-up system.

**Results:**

We improved the fatty alcohol production by systematically optimizing the fatty alcohol biosynthesis pathway, mainly targeting three key steps from fatty acyl-acyl carrier proteins (ACPs) to fatty alcohols, which are sequentially catalyzed by thioesterase, acyl-coenzyme A (CoA) synthase and fatty acyl-CoA reductase. By coexpression of thioesterase gene *BTE*, acyl-CoA synthase gene *fadD* and fatty acyl-CoA reductase gene *acr1*, 210.1 mg/L C12/14 alcohol was obtained. A further optimization of expression level of *BTE*, *fadD* and *acr1* increased the C12/14 alcohol production to 449.2 mg/L, accounting for 75.0% of the total fatty alcohol production (598.6 mg/L). In addition, by coexpression of thioesterase gene ‘*tesA*, acyl-CoA synthase gene *fadD* and fatty acyl-CoA reductase gene *FAR*, 101.5 mg/L C16/18 alcohol was obtained, with C16/18 alcohol accounting for 89.2% of the total fatty alcohol production.

**Conclusions:**

To our knowledge, this is the first report on selective production of C12/14 and C16/18 alcohols by microbial fermentation. This work achieved high-specificity production of both C12/14 and C16/18 alcohols. The encouraging 598.6 mg/L of fatty alcohols represents the highest titer reported so far. In addition, the 101.5 mg/L 89.2% C16/18 alcohol suggests an important breakthrough in C16/18 alcohol production. A more detailed optimization of the expression level of fatty alcohol biosynthesis pathway may contribute to a further improvement of fatty alcohol production.

## Background

Fatty alcohols, usually with hydrocarbon chains ranging from C8 to C18, are composed of a nonpolar, lipophilic carbon chain and a polar, hydrophilic hydroxyl group. The C8/10 alcohol (a blend of C8 and C10 alcohols) only has limited applications in the production of plasticizers, while the C12-C18 alcohols find applications in a multitude of uses. The C12/14 alcohol (a blend of C12 and C14 alcohols) can be used as lubricant additives, and the C16/18 alcohol (a blend of C16 and C18 alcohols) can be employed as defoamers, solubility retarders, and consistency giving factors. More importantly, the C12-C18 alcohols can be used to produce detergents and surfactants by replacing their hydroxy group with other larger hydrophilic groups [1,2]. Now the fatty alcohol market is dominated by natural alcohol and synthetic alcohol products. The natural alcohols are prepared from natural oil such as coconut oil and palm oil using transesterification and hydrogenation processes. The synthetic alcohols are produced from petrochemical feedstocks mainly using Ziegler-Process and Oxo-Process. However, these processes either need harsh production environments, or bring harmful materials to the environment. Besides, they are both now facing the huge challenge from the increasingly higher price of the raw materials. Therefore, increasing attentions have been paid to the microbial production of fatty alcohols from renewable resources [3,4].

**Figure 1:**
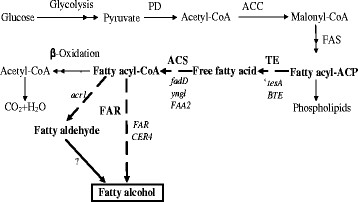
**Biosynthetic pathways of fatty alcohol.** The nodes we selected for metabolic engineering are marked in bold. The real lines represent the native pathways of *E. coli*, while the broken line shows a nonnative pathway of the host. The actual genes used for each step are shown in italics. PD: pyruvate dehydrogenase; ACC: acetyl-CoA carboxylase; FAS: fatty acid synthase; ACP: acyl carrier protein; TE: thioesterase (‘*tesA* and *BTE*); ACS: acyl-CoA synthase (*fadD*, *yngl* and *FAA2*); FAR: fatty acyl-CoA reductase (*FAR*, *acr1* and *CER4*).

The biosynthesis of fatty alcohols mainly employs the de novo fatty acid synthetic pathway of microbes. Fatty acyl-acyl carrier proteins (ACPs) are firstly manufactured via the fatty acid biosynthesis pathway. Then fatty acyl-ACPs are converted to free fatty acids (FFAs) and fatty acyl-coenzyme A (CoA) sequentially catalyzed by thioesterase and acyl-CoA synthase [5]. Next, the fatty acyl-CoAs are reduced to fatty aldehydes or fatty alcohols in a NADPH-dependent reaction catalyzed by fatty acyl-CoA reductase. The synthesized fatty aldehydes can be further converted to fatty alcohols by an unknown alcohol dehydrogenase or aldehyde reductase of *E. coli* (Figure 1) [6,7].

The thioesterase, acyl-CoA synthase and fatty acyl-CoA reductase play the key role in the fatty alcohol biosynthesis [3]. Either thioesterases, acyl-CoA synthases or fatty acyl-CoA reductases from different organisms have differences in substrate specificity [8]. For examples, the thioesterase BTE from *Umbellularia californica* prefers the medium-chain acyl-ACPs [9-11], and the thioesterase ‘TesA (a ‘leaderless’ version of TesA) from *E. coli* possesses a much broader substrate preference [3,5,12]. As for the fatty acyl-CoA synthase, the FadD from *E. coli* can efficiently activate the fatty acids ranging from C10 to C18, but FAA2 from *Saccharomyces cerevisiae* prefers the medium-chain fatty acids [13,14]. Many fatty acyl-CoA reductases, such as Acr1, FAR and CER4, are capable of converting fatty acyl-CoAs to fatty aldehydes (Acr1) or fatty alcohols (FAR and CER4) [6,15-18]. To investigate the substrate specificities of FAR and CER4, Doan *et al*. determined the composition of the fatty alcohols obtained by expressing a single FAR or CER4 in *E. coli *[19]. It was not a compellent strategy for evaluating the substrate specificity of fatty acyl-CoA reductase, since the FFAs with different chain lengths are not equally accumulated in the host. Though the substrate specificity of Acr1 was characterized *in vitro* by Reiser and Somervill [6], it is also needed to further characterize its substrate specificity *in vivo* to better simulate the cytocatalytic production of fatty alcohols.

Based on the achieved knowledge of the fatty alcohol biosynthesis pathway, microbial production of fatty alcohols was carried out in engineered *E. coli *[3]. Steen *et al*. enhanced the production of the medium chain fatty alcohols in an engineered *E. coli* by overexpression of the fatty alcohol biosynthesis pathway and deletion of the endogenous *fadE* gene (encoding acyl-CoA dehydrogenase) of *E. coli*. In addition, they made attempts to tailor the composition of fatty alcohols by employing thioesterases preferring different chain-length fatty acyl-ACPs. The C12 alcohol was mainly produced by expressing *UcFatB,* while expression of *ChFatB3* or ‘*tesA* mainly contributed to production of C14 alcohol. However, only a small proportion of C16/18 alcohol was observed in their engineered strains [3].

Though fatty alcohols have been produced by engineered *E. coli*, however, several problems have not been solved in previous studies. First, no improvement has been achieved in the production of C16/18 alcohol; Second, detailed optimization of the fatty alcohol biosynthesis pathway has not been carried out; Third, the performance of the engineered *E. coli* on the production of fatty alcohols has not been investigated in scaleable fermentation processes.

In order to optimize the fatty alcohol biosynthesis pathway for selectively enhanced production of C12/14 and C16/18 alcohols, we investigated the effect of acyl-CoA synthases and fatty acyl-CoA reductases on the production of fatty alcohols, determined the C12/14 and C16/18 alcohol production across different thioesterases, acyl-CoA synthases and fatty acyl-CoA reductases, optimized the expression level of associated enzymes, and evaluated the biocatalytic properties of the engineered strains in fed-batch fermentation.

## Results

### Fatty alcohol distribution in engineered strains employing fatty acyl-CoA reductases FAR, Acr1 or CER4

To employ the proper fatty acyl-CoA reductases for selective production of C12/14 and C16/18 alcohols, we determined the fatty alcohol distribution of the engineered strains Zh05, Zh07 and Zh08 by adding an equivalent blend of lauric acid (C12:0), myristic acid (C14:0), palmitic acid (C16:0), stearic acid (C18:0) and oleic acid (C18:1) (200 mg/L each) into their cultures. Zh05, Zh07, and Zh08 overexpressed *FAR* gene from *S. chinensisacr1* gene from *A. baylyi* and *CER4* gene from *A. thaliana*, respectively. To promote the conversion of FFAs to fatty acyl-CoAs, an endogenous acyl-CoA synthase gene (*fadD*) was also coexpressed in all three engineered strains. Given FadD has close substrate specificities towards C10 to C18 FFAs [13], the substrate preferences of CER4 and FAR can be roughly speculated according to the chain length distributions of the synthesized fatty alcohols. Though the substrate specificity of Acr1 can not be concluded from the distribution of fatty alcohols, the combined effect of Acr1 and endogenous alcohol dehydrogenase/aldehyde reductase on the fatty alcohol production can be clearly determined with the expression of Acr1 in *E. coli*. In addition, the endogenous FFAs of the host were determined in the same conditions where FFAs are supplied in the culture. The host *E. coli* BL21(DE3) only produced 15.7 mg/L FFAs (C12, 1.1 mg/L; C14, 2.0 mg/L; C16, 7.2 mg/L; C18, 5.4 mg/L), and the engineered strains Zh05, Zh07 and Zh08 produced nearly the same endogenous FFAs. Therefore, the endogenous FFAs of the hosts can be almost neglected when compared with fatty acids exogenously supplemented.

**Figure 2:**
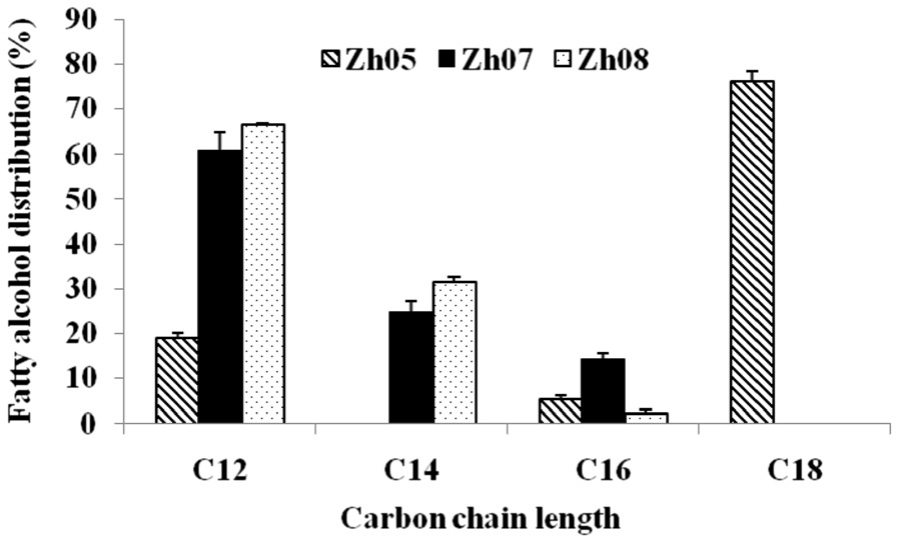
**Fatty alcohol distribution in engineered strains employing fatty acyl-CoA reductases FAR, Acr1 or CER4.** The distribution was assayed by measuring the formation of fatty alcohols in the cultures of Zh05, Zh07 and Zh08, which were all supplemented with an equivalent blend of lauric acid, myristic acid, palmitic acid, stearic acid and oleic acid ahead of induction. *FAR*, *acr1* and *CER4* are fatty acyl-CoA reductase genes from *S. chinensis*, *A. baylyi* and *A. thaliana*, respectively; *fadD*, acyl-CoA synthase gene from *E. coli*; Zh05, employing *FAR* and *fadD* genes; Zh07, employing *FAR* and *fadD* genes; Zh08, employing *CER4* and *fadD* genes. The error bars represent the range from two independent experiments.

Fatty alcohol production in these engineered strains was analyzed by GC-MS. Zh07 and Zh08 produced a predominant C12/14 alcohol, 85.8% and 97.7%, respectively, with nearly no C18 alcohol produced, while Zh05 produced 81.1% C16/18 alcohol, with C18 alcohol accounting for 75.9% (Figure 2). That is to say, when combined the expression of FadD with Acr1 or CER4, the engineered strains produced a predominant C12/14 alcohol, and when combined the expression of FadD with FAR, the engineered strain produced a predominant C16/18 alcohol. It demonstrates that CER4 prefers acyl group with relatively shorter carbon chain lengths, while FAR prefers a longer acyl group, especially the oleoyl group. The expression of Acr1 contributed to the synthesis of C12/14 alcohol, with the help of endogenous alcohol dehydrogenase/aldehyde reductase. It was worth mentioning that Zh05, Zh07 and Zh08 produced neglectable stearyl alcohol, and the C18 alcohol produced was predominately oleyl alcohol. In addition, Zh07 produced 39.6-fold more C12/14 alcohol than Zh08, suggesting expression of Acr1 in *E. coli* is capable of producing more C12/14 alcohol than expression of CER4.

### Comparison of the efficiencies of three different acyl-CoA synthases on fatty alcohol production

As above mentioned, the desired fatty acyl-CoA reductases have been obtained. And the substrate specificities of thioesterases BTE and ‘TesA have been well characterized. BTE produces a predominant C12/14 FFAs, and ‘TesA mainly produces C14-C18 FFAs. To better activate the specific FFAs to corresponding fatty acyl-CoAs, it is necessary to overexpress a proper acyl-CoA synthase. Therefore, we compared the efficiencies of three different acyl-CoA synthases (FadD, Yngl and FAA2) on the production of C12/14 and C16/18 alcohols from glucose.

**Figure 3:**
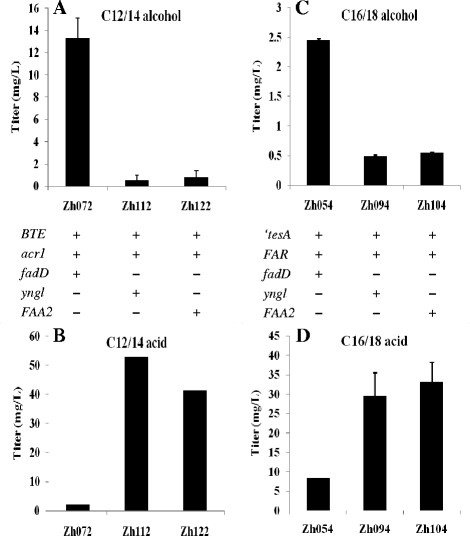
**The efficiencies of FadD, Yngl and FAA2 on the production of fatty alcohols and the activation of FFAs.** FadD, Yngl and FAA2 are acyl-CoA synthases from *E. coli*, *B. subtilis* and *S. cerevisiae*, respectively. A, B, the C12/14 alcohol production (**A**) and the C12/14 acid unutilized (**B**) by employing FadD, Yngl or FAA2 as the acyl-CoA synthase. C, D, the C16/18 alcohol production (**C**) and the C16/18 acid unutilized (**D**) by employing FadD, Yngl or FAA2 as the acyl-CoA synthase. The error bars represent the range from two independent experiments.

**Figure 4:**
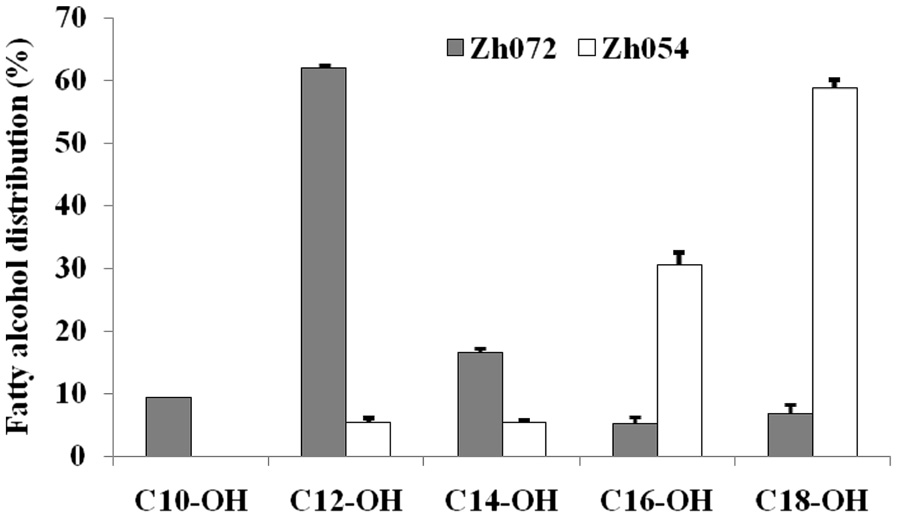
**Fatty alcohol composition of Zh072 and Zh054 cultures in shake flask.** Filled columns are for Zh072 (coexpression of Acr1, FadD and BTE); Open columns are for Zh054 (coexpression of FAR, FadD and ‘TesA). The error bars represent the range from two independent experiments.

When coexpressed with BTE and Acr1, the strain with FadD overexpressed (Zh072) produced 13.3 mg/L C12/14 alcohol in shake flask, 23.6-fold and 16.8-fold higher than the strains employing Yngl from *B. subtilis* (Zh112) and FAA2 (Zh122) from *S. cerevisiae*, respectively (Figure 3A). In addition, Zh072 produced C12/14 alcohol as its major fatty alcohol constituent (78.6%), with C10 alcohol (9.3%), C16 alcohol (5.2%) and C18 alcohol (6.9%) being the other fatty alcohols observed (Figure 4).

To further confirm that acyl-CoA synthases contributed to the difference in the production of C12/14 alcohol, we determined the FFAs unutilized by these engineered strains. In contrast to 52.7 mg/L and 41.3 mg/L C12/14 acid unutilized by Zh112 and Zh122, respectively, only 2.1 mg/L C12/14 acid was not utilized by Zh072 (Figure 3B). It demonstrated that FadD could activate C12/14 acid to C12/14 acyl-CoA much more efficiently than Yngl and FAA2, and the higher reaction rate of FadD contributed to Zh072 achieving a higher C12/14 alcohol titer than Zh112 and Zh122.

We then compared their efficiencies on the production of C16/18 alcohol. When coexpressed with ‘TesA and FAR, the strain with FadD overproduction (Zh054) produced 2.45 mg/L C16/18 alcohol in shake flask, 4.95-fold and 4.44-fold higher than that of the strains employing Yngl (Zh094) and FAA2 (Zh104), respectively (Figure 3C). This result illuminated that, compared with Yngl and FAA2, FadD could direct more C16/18 acid to corresponding acyl-CoA, which was further reduced to C16/18 alcohol by FAR. This conclusion could be further confirmed by the analysis of the FFAs unutilized. Only 8.3 mg/L C16/18 acid was unutilized by Zh054, in contrast to 29.5 mg/L and 33.1 mg/L C16/18 acid unutilized by Zh094 and Zh104, respectively (Figure 3D).

We also analyzed the composition of fatty alcohols synthesized by Zh054. Zh054 produced C16/18 alcohol as its predominant fatty alcohol constituent (89.2%), with C12 alcohol (5.4%) and C14 alcohol (5.4%) being the other fatty alcohols observed (Figure 4).

### Optimization of the expression level of C12/14 alcohol biosynthesis pathway

Thus far, we have obtained two engineered strains Zh072 and Zh054, which were well performed in high-specificity production of C12/14 alcohols and C16/18 alcohols, respectively. However, it is possible to further enhance the production of fatty alcohols in *E. coli* by optimizing the expression level of fatty alcohol biosynthesis pathway. Given C12/14 alcohol and C16/18 alcohol biosynthesis pathways are quite similar to each other, we only did the optimization of the expression level of C12/14 alcohol biosynthesis pathway.

**Figure 5:**
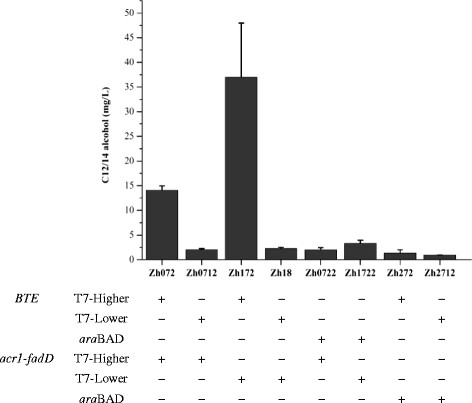
**Optimization of the expression level of C12/14 alcohol biosynthesis pathway.** Plasmids with different copy numbers and promoters are used to optimize the expression level of C12/14 alcohol biosynthesis pathway. Zh172, with *BTE* and *fadD*-*acr1* being carried by a higher copy number plasmid (T7 promoter) and a lower copy number plasmid (T7 promoter), respectively, achieved the highest C12/14 alcohol titer. Zh072 using two higher copy number plasmids (T7 promoter) accumulated a medium amount of C12/14 alcohol. Other strains produced a low concentration of C12/14 alcohol. The error bars represent the range from two independent experiments.

Firstly, several plasmids with different copy numbers were employed to bear *BTE* and *fadD*-*acr1*. When *BTE* and *fadD*-*acr1* were carried by a higher copy number plasmid and a lower copy number plasmid, respectively, corresponding strain Zh172 achieved the highest C12/14 alcohol titer. Zh072 using two higher copy number plasmids accumulated a 2.6-fold lower titer than Zh172. Zh0712 and Zh18, both carrying a lower copy number *BTE*, produced much lower C12/14 alcohol titers when compared with Zh072 and Zh172 (Figure 5).

The genes above mentioned are all under the control of the strong T7 promoter. Given Lennen *et al.* achieved a high production of fatty acids by using a medium-strength *ara*BAD promoter (P_BAD_) [20], we thus partly replace the T7 promoter with P_BAD_. But unfortunately, none of the engineered strains improved the production of C12/14 alcohol, when either *BTE* or *acr1* gene was under the control of P_BAD_ (Figure 5).

### Fed-batch fermention

**Figure 6:**
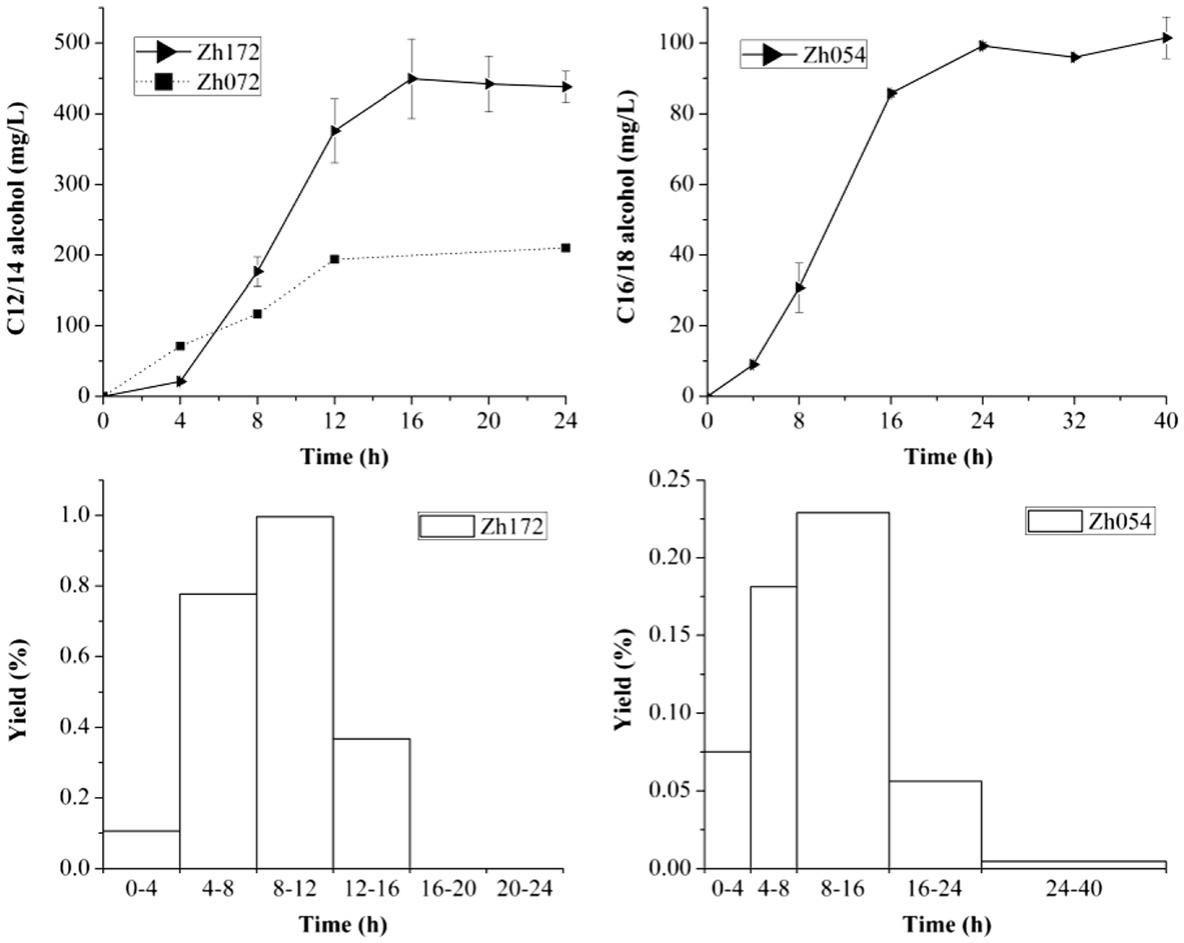
**Characterization of fatty alcohol production of Zh172, Zh072 and Zh054 in the fed-batch fermentation.** A, B, the production of C12/14 alcohol (A) and C16/18 alcohol (B) in fed-batch fermentations; C, D, the yield (g product/100 g glucose) of Zh172 (C) and Zh054 (D). The error bars represent the range from two independent experiments.

To evaluate the fatty alcohol production in a scaleable process, fed-batch fermentations of Zh172, Zh072 and Zh054 were carried out at 5-L scale. Over the course of the fermentation, Zh172 produced 598.6 mg/L fatty alcohols, with C12/14 alcohol accounting for 75.0% (449.2 mg/L), while Zh054 accumulated 101.5 mg/L C16/18 alcohol. The C12/14 alcohol titer of Zh172 was 2.1 folds higher than that of Zh072 (210.1 mg/L) (Figure 6). For Zh072, the C12/14 alcohol productivity was relatively constant at 15.6 mg/L/h just after the protein production was triggered with IPTG. However, the linear C12/14 alcohol productivity of Zh172 and C16/18 alcohol productivity of Zh054 appeared approximately 4 h post-induction, constant at 35.7 mg/L/h and 6.5 mg/L/h, respectively. In addition, a maximal C12/14 alcohol yield of 1.0% (i.e. 0.01 g C12/14 alcohol/g carbon source consumed) and a maximal C16/18 alcohol yield of 0.23% were observed during the post-induction, linear fatty alcohol production period (Figure 6).

It was worth noting that, at the end of the fermentation, 621.1 mg/L C16/18 FFA and 133.4 mg/L C12/14 FFA were unutilized by Zh054 and Zh072, respectively. Contrastively, only 316.0 mg/L and 39.4 mg/L corresponding FFAs were unutilized by Zh054 and Zh072 at the 8-h time point, when both Zh054 and Zh072 produced fatty alcohols at a maximum productivity. It demonstrated that the produced FFAs were no longer converted to fatty alcohols in the later stage of the fermentation. The fatty alcohol production ceased ahead of the production of FFAs.

## Discussion

Expression of alcohol-forming fatty acyl-CoA reductases in *E. coli* can result in the biosynthesis of fatty alcohols from endogenous *E. coli* fatty acids, but the levels were quite low [19]. To improve the production of fatty alcohols, Steen *et al*. carried out a further genetic modification of *E. coli*, and achieved an increased titer (~60 mg/L) of the medium chain fatty alcohols (C12 or C14 alcohols) [3]. In their strategy, they employed thioesterases with different substrate specificities to tailor the composition of the FFAs, and used the aldehyde-forming fatty acyl-CoA reductase Acr1 for the conversion of fatty acyl-CoAs to fatty aldehydes. The synthesized fatty aldehydes can be further converted to fatty alcohols by an unknown alcohol dehydrogenase/aldehyde reductase of *E. coli *[6].

However, Steen *et al*. just obtained a small quantity of C16/18 alcohol, though they used the thioesterase ‘TesA, which was capable of yielding a large proportion of C16/18 FFA [3]. As aforementioned, expression of Acr1 in *E. coli* can only obtain a predominant C12/14 alcohol, even if the longer FFAs are supplied. Therefore, their poor C16/18 alcohol production is probably attributed to the Acr1 they employed. However, Reiser and Somerville spectroscopically assayed the substrate specificity of an unpurified Acr1 protein by measuring the acyl-CoA-dependent oxidation of NADPH, and found Acr1 had the biggest substrate preference towards C16/18 acyl-CoAs [6]. It suggests that the endogenous alcohol dehydrogenase/aldehyde reductase of *E. coli* may have substrate preference for fatty aldehydes with shorter chain lengths, and thus block the conversion of C16/18 aldehydes to corresponding alcohols. This bioconversion process will be much clearer if the purified Acr1 protein can be characterized by determining its synthesized products.

We are cognizant of the fact that it is impossible to obtain all our desired fatty alcohols just by tailoring the composition of FFAs. We thus replaced Acr1 with FAR as the fatty acyl-CoA reductase for the production of long chain fatty alcohols, given FAR preferred the longer acyl groups. To data, no research was performed to optimize the long chain fatty alcohol biosynthesis pathway. Therefore, we combined the expression of thioesterase and acyl-CoA synthase with FAR to enhance the long chain fatty alcohol production from glucose. Our achieved titer of 101.5 mg/L represents the highest C16/18 alcohol production ever reported [4,19].

In addition, Steen *et al*. found the importance of acyl-CoA synthase in improving the fatty alcohol production [3]. It is of significance to explore and find a proper acyl-CoA synthase that capable of enhancing the production of C12/14 or C16/18 alcohol. We found FadD possessed broad substrate specificity and high catalytic activity, based on the investigation of three different acyl-CoA synthases from *E. coli* (FadD), *B. subtilis* (Yngl) and *S. cerevisiae* (FAA2), respectively. FadD can convert most of the C12-C18 FFAs into their activated forms – fatty acyl-CoAs, and it was suitable for the production of both C12/14 and C16/18 alcohols.

The level of free CoA may also play an important role in the conversion of FFAs to fatty acyl-CoAs, since free CoA directly participates the reaction as a substrate. Once the fatty acyl-CoAs accumulate, that will lead to a reduction of free CoA. The decreased level of free CoA may further block the activation of FFAs. In contrast, increased level of free CoA will benefit the conversion of FFAs to fatty acyl-CoAs.

With a series of combination across different thioesterases, acyl-CoA synthases and fatty acyl-CoA reductases, we constructed two engineered strains (Zh072 and Zh054) that were capable of high-specificity production of C12/14 and C16/18 alcohols, respectively. Lennen *et al*. achieved a high level of fatty acid production by using a medium-strength P_BAD _[20]. Therefore, it is possible to enhance the fatty alcohol production by optimization of the expression level of fatty alcohol biosynthesis pathway. No enhanced fatty alcohol production was obtained when either *BTE* or *acr1* was under the control of P_BAD_. However, an obviously improved fatty alcohol production was achieved by optimization of the plasmid copy number. This result provides a useful clue for enhancing the fatty alcohol production. Of course, a more detailed optimization of the expression level is still needed to further improve the production of fatty alcohols.

Given no process data is available on the fatty alcohol production by engineered *E. coli*, we evaluated the performances of three well-performed strains in the fed-batch fermentation. The maximum fatty alcohol productivity was observed in the early stage of the post-induction. Over this phase, fatty alcohol production ceased. Given FFAs can be also converted to fatty aldehydes [6,21-23], maybe the released FFAs were predominately transformed to fatty aldehydes instead of fatty alcohols in the latter stage. To make the fermentation processes be clearer, it is needed to perform some further investigations focusing on the production of fatty aldehydes.

The lower titer in our shake-flask study was probably attributed to the low buffering capacity of our culture medium, whose pH decreased rapidly with the growth of cells. The resultant lower biomass caused the decreased fatty alcohol production. The fatty alcohol production dramatically increased in the fed-batch fermentation with pH adaption.

In addition, given that fatty acyl-CoA reductases need the participation of coenzyme NADPH [17], the production of fatty alcohols may be enhanced by expressing the NADPH regeneration system such as phosphite dehydrogenase and glucose-6-phosphate dehydrogenase in *E. coli*.

## Conclusions

Given the importance of the C12-C18 fatty alcohols as surfactants and the issues associated with petroleum and natural oil feedstocks, increasing attention is being paid to seeking after the low-cost renewable routes from sugars. In addition, it will have a better downstream application to obtain the fatty alcohol products with narrow-range distribution of carbon chain lengths. Therefore, the ultimate objective of this study was to realize the high-level and selective production of C12/14 and C16/18 alcohols in engineered *E. coli* by optimization of the fatty alcohol biosynthesis pathway. To our knowledge, this is the first report on selective production of C12/14 and C16/18 alcohols in engineered microbes. High-specificity production of both C12/14 and C16/18 alcohols has been obtained. The C12/14 alcohol and C16/18 alcohol occupied 75.0% and 89.2% of total fatty alcohols produced by Zh172 and Zh054, respectively. Without detailed optimization of the production conditions, an encouraging 598.6 mg/L of fatty alcohols was finally achieved, representing the highest fatty alcohol production achieved so far. In addition, the 101.5 mg/L 89.2% C16/18 alcohol suggests an important breakthrough in C16/18 alcohol production. A more detailed optimization of the expression level of fatty alcohol biosynthesis pathway may contribute to a further improvement of fatty alcohol production. Our study provides the groundwork for microbial production of a surfactant range of fatty alcohols from the rich lignocellulose sources.

## Methods

### Bacterial strains, media, and growth conditions

The bacterial strains used in this study are listed in Table 1. *E. coli* BL21(DE3) (Invitrogen, Carlsbad, CA) was used as the host to overproduce proteins. During strain construction, cultures were grown aerobically at 37°C in Luria Broth (10 g/L tryptone, 10 g/L NaCl, and 5 g/L yeast extract). Kanamycin (50 mg/L) or chloramphenicol (34 mg/L) was added if necessary. For initial production experiments in shake flasks, strains were grown in a M9 medium (15.13 g/L Na_2_HPO_4_·12H_2_O, 3 g/L KH_2_PO_4_, 1 g/L NH_4_Cl, 0.5 g/L NaCl, 2 mM MgSO4) containing 20 g/L of glucose or 20 g/L of glycerol. The engineered strains were fed with glycerol as carbon source if they carried the recombinant plasmids using *ara*BAD promoter, otherwise they were fed with glucose. Another 0.9% beef extract was added to the M9 medium for the conversion of exogenous fatty acids to fatty alcohols. Protein production was induced with 0.5 mM isopropyl β-D-thiogalactoside (IPTG) at 30°C. For the fed-batch fermentation in a 5 L BIOSTAT® B plus fermentor (Sartorius Stedim Biotech GmbH, Goettingen, Germany), strains were grown in the defined batch medium consisted of the following: 7.5 g/L K_2_HPO_4_·3H_2_O, 2.1 g/L citric acid monohydrate, 0.3 g/L ferric ammonium citrate, 2.92 g/L (NH4)_2_SO_4_, 2 mM MgSO_4_, trace metals mix (2.86 mg/L H_3_BO_3_, 1.81 mg/L MnCl_2_·4H_2_O, 0.222 mg/L ZnSO_4_·7H_2_O, 0.39 mg/L Na_2_MoO_4_·2H_2_O, 0.079 mg/L CuSO_4_·5H_2_O, 49.4 μg/L Co(NO_3_)_2_·6H_2_O). The fermentation temperature was controlled at 30°C and the pH at 7.0. The pH was maintained using NH_3_·H_2_O. Cells were induced at an OD_600_ of ~15 using 0.5 mM IPTG. The glucose feed solution was continuously added into the cultures at the rate of 3 ~ 5 g/L/h, and the residual glucose in the cultures was maintained at 0.1 ~ 0.3 g/L.

**Table 1:** Bacterial strains and plasmids used in this study

**Plasmid or strain**	**Relevant genotype**	**Reference or source**
Plasmids		
pET-30a(+)	pBR322 *ori lacI* T7*lac* Kan^r^	Novagen
pACYCDuet-1	P15A *ori lacI* T7*lac* Cm^r^	Novagen
pCOLADuet-1	ColA *ori lacI* T7*lac* Kan^r^	Novagen
pBAD/*Myc*-HisA	pBR322 *ori ara*BAD Amp^r^	Invitrogen
pZh05	pACYCDuet-1 harboring *FAR* from Jojoba and *fadD* from *E. coli*	This study
pZh07	pACYCDuet-1 harboring *acr1* from *A. baylyi* and *fadD* from *E. coli*	This study
pZh08	pACYCDuet-1 harboring *CER4* from *A. thaliana* and *fadD* from *E. coli*	This study
pZh09	pACYCDuet-1 harboring *FAR* from Jojoba and *yngl* from *B. subtilis*	This study
pZh10	pACYCDuet-1 harboring *FAR* from Jojoba and *FAA2* from *S. cerevisiae*	This study
pZh11	pACYCDuet-1 harboring *acr1 from A. baylyi* and *yngl* from *B. subtilis*	This study
pZh12	pACYCDuet-1 harboring *acr1* from *A. baylyi* and *FAA2* from *S. cerevisiae*	This study
pZh17	pCOLADuet-1 harboring *acr1* from *A. baylyi* and *fadD* from *E. coli*	This study
pZh18	pCOLADuet-1 harboring *acr1* from *A. baylyi*, *fadD* from *E. coli* and *BTE* from *U. californica*	This study
pZh27	pBAD/*Myc*-HisA harboring *acr1* from *A. baylyi* and *fadD* from *E. coli*	This study
pYN2	pET-30a(+) harboring *BTE* from *U. californica*	26
pLL4	pCOLADuet-1 harboring *‘tesA* from *E. coli*	This study
pYN12	pCOLADuet-1 harboring *BTE* from *U. californica*	This study
pXW2	pACYCDuet-1 harboring *BTE* from *U. californica*	This study
pYN22	pBAD/*Myc*-HisA harboring *BTE* from *U. californica*	This study
Strains		
DH5α	F^-^,φ80d*lacZ*ΔM15, Δ(*lacZYA-argF*)U169, *deoR*,recA1, endA1, hsdR17(rk^-^,mk^+^), phoA, supE44, λ-,thi^-^1, gyrA96, relA1	Takara
BL21 (DE3)	F^-^*ompT gal dcm lon hsdSB(r*_*B*_^*-*^*m*_*B*_^*-*^*)* λ(DE3)	Invitrogen
Zh05	*E. coli* BL21 (DE3) bearing pZh05	This study
Zh07	*E. coli* BL21 (DE3) bearing pZh07	This study
Zh08	*E. coli* BL21 (DE3) bearing pZh08	This study
Zh072	*E. coli* BL21 (DE3) bearing pZh07 and pYN2	This study
Zh112	*E. coli* BL21 (DE3) bearing pZh11 and pYN2	This study
Zh122	*E. coli* BL21 (DE3) bearing pZh12 and pYN2	This study
Zh054	*E. coli* BL21 (DE3) bearing pZh05 and pLL4	This study
Zh094	*E. coli* BL21 (DE3) bearing pZh09 and pLL4	This study
Zh104	*E. coli* BL21 (DE3) bearing pZh10 and pLL4	This study
Zh0712	*E. coli* BL21 (DE3) bearing pZh07 and pYN12	This study
Zh172	*E. coli* BL21 (DE3) bearing pZh17 and pXW2	This study
Zh18	*E. coli* BL21 (DE3) bearing pZh18	This study
Zh0722	*E. coli* BL21 (DE3) bearing pZh07 and pYN22	This study
Zh1722	*E. coli* BL21 (DE3) bearing pZh17 and pYN22	This study
Zh272	*E. coli* BL21 (DE3) bearing pZh27 and pXW2	This study
Zh2712	*E. coli* BL21 (DE3) bearing pZh27 and pYN12	This study

### Regents

The arachidyl alcohol and arachidic acid were ordered from Acros Organics (Geel, Belgium) and Alfa Aesar (Ward Hill, MA), respectively. All restriction enzymes and T4 DNA ligatase were purchased from Fermentas (Vilnius, Lithuania). The *Pyrobest* DNA polymerase was supplied by Takara Biotechnology (Dalian, China). The pACYCDuet-1, pCOLADuet-1 and pET-30a(+) expression vectors were purchased from Novagen (Darmstadt, Germany). Oligonucleotides were ordered from BGI (Beijing, China).

### Plasmid construction

Genes derived from *E. coli* str. K12 substr. MG1655 (*fadD* [Genbank: 946327], and ‘*tesA*, a leaderless version of *tesA* [Genbank: 945127]) [24,25], *Saccharomyces cerevisiae* S288c (*FAA2* [Genbank: NM_001178906]), *Bacillus subtilis* ATCC 23857 (*yngl* [Genbank: 939955]), and *Acinetobacter baylyi* ATCC 33305 (*acr1* [Genbank: U77680]) were obtained by polymerase chain reaction (PCR) using *Pyrobest* DNA polymerase. The *CER4* gene [Genbank: NM_119537] was amplified by PCR using the total cDNA of *Arabidopsis thaliana* as the template. The *BTE* gene [Genbank: M94159] of *Umbellularia californica* and a codon-optimized *FAR* gene of *Simmondsia chinensis* [Genbank: JQ768345] were chemically synthesized. Custom oligonucleotides (primers) for all PCR amplifications are shown in Table 2.

**Table 2:** Primers used in this study

**Gene**	**Primer name**	**Sequence (5'→ 3')**
*BTE*	BTE-NdeF	GGAATTCCATATGGCCACCACCTCTTTAG
	BTE-NotR	ATAAGAATGCGGCCGCTTACACCCTCGGTTCTGCG
	BTE-NcoF	CTAGCCATGGCCACCACCTCTTTAGC
	BTE-XhoR	CACCTCGAGTTACACCCTCGGTTCTGCG
	T7min-BamF	CAGGGATCCTAATACGACTCACTATAGG
*‘tesA*	tesA-NcoF	CATGCCATGGCGGACACGTTATTGATTCTG
	tesA-EcoR	CAGGAATTCTTATGAGTCATGATTTACTAAAG
*fadD*	fadD-BglF	CTCAGATCTCATGAAGAAGGTTTGGCTTAACC
	fadD-XhoR	CACCTCGAGTTATCAGGCTTTATTGTCCACTTTG
*yngl*	yngl-Eco32F	CACGATATCGATGGCTGAACTCATCCATTCC
	yngl-XhoR	CACCTCGAGTCATTGACATGATGATAAGTTG
*FAA2*	FAA2-NdeF	GGAATTCCATATGGCCGCTCCAGATTATGCAC
	FAA2-Eco32R	CACGATATCTTACTAAAGCTTTTCTGTCTTGACTAG
*FAR*	FAR-NcoF	CATGCCATGGAGGAAATGGGCAGC
	FAR-BamR	CGGGATCCTTAGTTAAGAACGTGCTCCAC
*CER4*	CER4-NcoF	CATGCCATGGGCATGTCGACAGAAATGGAGGTCG
	CER4-BamR	CGGGATCCTTAGAAGACATACTTAAGCAGCCC
*acr1*	acr1-NcoF	CATGCCATGGTGAACAAAAAACTTGAAGC
	acr1-BamR	CGGGATCCTTACCAGTGTTCGCCTG

Underlines indicate restriction enzyme sites.

The amplified *FAR* gene was firstly gel purified, then digested with NcoI and BamHI, and finally ligated into pACYCDuet-1 that had been digested with the same restriction enzymes, yielding pZh01. In the same means, the BglII-XhoI digested *fadD*, NdeI-EcoRV digested *FAA2* and EcoRV-XhoI digested *yngl* were ligated into pZh01 to yield pZh05, pZh09 and pZh10, respectively. For the construction of pZh07 and pZh08, the *FAR* located at pZh05 was replaced with *acr1* and *CER4*, respectively. To yield pZh11 and pZh12, the *acr1* gene was removed from the plasmid pZh07 by restriction enzyme digestion with NcoI/EcoRI, gel purified, and ligated into pZh09 and pZh10 whose *FAR* gene had been removed by restriction enzyme digestion. To create pZh17 and pZh27, *acr1fadD*, cut from pZh07 with NcoI/XhoI, was cloned into corresponding sites of pCOLADuet-1 and pBAD/*Myc*-HisA, respectively. Three sets of primers, BTE-NdeF/BTE-NotR, BTE-NdeF/BTE-XhoR and BTE-NcoF/BTE-XhoR, were used to amplify the thioesterase gene *BTE*. The amplified BTEs were inserted into the NdeI/NotI site of pET-30a(+), NdeI/XhoI site of pCOLADuet-1 and pACYCDuet-1, and NcoI/XhoI site of pBAD/*Myc*-HisA, respectively. The resulted recombinant plasmids were designated as pYN2 [26], pYN12, pXW2 and pYN22, respectively. A primer set of T7min-BamF/BTE-NotR was used to amplify the *BTE* with a T7 promoter (*T7BTE*) using pYN2 as the template, the PCR product was digested with BamHI/NotI, and then inserted into pZh17 to yield pZh18. As for the thioesterases gene ‘*tesA*, the NcoI/EcoRI digested *‘tesA* were ligated into corresponding sites of pCOLADuet-1, creating pLL4. Plasmids used in this work are listed in Table 1.

### Detection of metabolites

The harvested cultures were first heated with boiled water for 20 minutes to partly lyse the cells. Then the pH values of the cultures were adjusted to 2.0 using hydrochloric acid. Next chloroform–methanol (v/v, 2:1) was added into the cultures. The resultant blends were vortexed for a few minutes and then left overnight to extract the fatty alcohols and fatty acids. The arachidyl alcohol and arachic acid were added into the cultures and used as the internal standards for the analysis of fatty alcohols and fatty acids, respectively. The desired products of the shake-flask culture were quantified by gas chromatography–mass spectrometry (GC-MS) using an Agilent 7890A system equipped with a HP-INNOWax (30 m × 0.25 mm; 0.25 μm film thickness). Helium was used as the carrier gas. The injection port was held at 250°C, and the following oven temperature program was carried out: 100°C for 2 min, increase of 10°C/min to 240°C for 15 min. The desired products of the fermentor culture were quantified by a gas chromatograph (GC) equipped with a flame ionization detector. The separation of FFAs was performed using a CP-FFAP CB capillary column (25 m × 0.25 mm; 0.2 μm film thickness) purchased from Agilent Technologies (Santa Clara, CA). The oven temperature was initially held at 100°C for 1 min, then raised with a gradient of 10°C/min until reaching 250°C, and finally held for 10 min. Nitrogen was used as the carrier gas. The injector and detector were held at 270°C and 300°C, respectively.

## Abbreviations

ACP: Acyl carrier protein; CoA: Coenzyme A; FFA: Free fatty acid; IPTG: Isopropyl β-D-thiogalactoside; PCR: Polymerase chain reaction; GC: Gas chromatography; GC-MS: Gas chromatography–mass spectrometry.

## Competing interests

The authors declare that they have no competing interests.

## Authors’ contributions

MX and GZ developed the idea for the study. YZ designed the research. YZ did the literature review and prepared the manuscript. MX and GZ helped to revise the manuscript. YZ and LL did the majority of the lab work, plasmid construction, strain cultivation and product detection. QL participated in the plasmid construction and product detection. YZ, JY and HL performed the fed-batch fermentation. JY, XW, WL and XX participated in the product detection. All authors read and approved the final manuscript.
